# Clinical features of gingivostomatitis due to primary infection of herpes simplex virus in children

**DOI:** 10.1186/s12879-020-05509-2

**Published:** 2020-10-20

**Authors:** Chen-Wei Huang, Chi-Hsien Hsieh, Ming-Ru Lin, Yhu-Chering Huang

**Affiliations:** 1 Department of Medicine, Chang Gung University College of Medicine, Taoyuan, Taiwan; 2 Department of Pediatrics, Chang Gung Memorial Hospital, No.5, Fu-Hsin Street, Kweishan, 333 Taoyuan, Taiwan

**Keywords:** Herpes simplex virus, Primary herpetic gingivostomatitis, Children, Clinical manifestation

## Abstract

**Background:**

Primary herpetic gingivostomatitis (PHGS) in children, though usually self-limited, might mimic bacterial and enteroviral pharyngitis clinically. We conducted a study to define the clinical features of PHGS in children.

**Methods:**

Between January 2012 and December 2016, 282 inpatients aged less than 19 years with cell culture-confirmed herpes simplex virus (HSV) infection in a medical center were identified from the virologic laboratory logbook. Clinical data were retrospectively collected.

**Results:**

Among the 282 inpatients, 185 cases were considered as PHGS and were included for analysis. Fever was present in 99.5%. The mean duration of fever was 5.11 days (±2.24) with the longest being 17 days. Common oral manifestations included oral ulcers (84.3%), which equally resided in the anterior and posterior part of the oral cavity (65.4% vs. 63.2%), gum swelling and/or bleeding (67.6%), and exudate coated tonsils (16.8%). Leukocytosis (WBC count > 15,000/uL^3^) was noted in 52 patients (28.1%) and a serum C-reactive protein level > 40 mg/L in 55 patients (29.7%). Fixty-five patients (35%) were diagnosed with PHGS on admission and were significantly more likely to have ulcers over the anterior oral cavity (76.1% vs. 26.7%) and gum swelling/bleeding (76.2% vs. 7.5%, *p*-value all < 0.001) on admission and were significantly less likely to receive antibiotic treatment (16.9 vs. 36.7%, *p*-value < 0.01) than others. Forty-six patients (25%) undiagnosed as PHGS on discharge were significantly more likely to have exudate coated on the tonsils, to receive antibiotic treatment and significantly less likely to have gum swelling/bleeding and oral ulcers (all *p*-values < 0.01).

**Conclusions:**

Meticulously identifying specific oral manifestations of gum swelling/bleeding and ulcers over the anterior oral cavity in children can help making the diagnosis of PHGS earlier and subsequently reduce unnecessary prescription of antibiotics.

## Background

Herpes simplex virus (HSV) belongs to the alpha-herpesviridae family, can be divided into two common pathogens, HSV-1 and HSV-2, and infects the humans [1–4]. Children infected with HSV may manifest many non-specific and systemic symptoms, including fever, headache, irritability, anorexia, and malaise. Many parents may attribute these symptoms to teething [5, 6]. Among the HSV-infected related diseases in children, primary herpetic gingivostomatitis (PHGS) is the most representative clinical manifestation in a proportion of around 13–30% [7, 8]. Although PHGS is usually a self-limited disease, some severe systemic complications have been reported, such as central nervous system (CNS) dysfunction [9, 10].

In Taiwan, human non-polio enteroviruses (NPEVs) circulate in summer and fall seasons with an epidemic of enterovirus A71 (EV-A71) occurring every 3–4 years [11, 12]. In children with NPEV infection, EV-A71 is a more concerning issue since it may rapidly develop neurologic and systemic complications in a small percentage of patients [13]. The clinical symptoms/signs of enteroviral infection include high fever and oral ulcers, which are similar to initial presentations of PHGS and may lead to a clinical dilemma to differentiate these two infectious disease entities at the first glance. The misdiagnosis of NPEV infection from PHGS may sometimes lead to unnecessary medical costs due to hospitalization for close observation of dangerous signs of severe enteroviral infections. Moreover, the duration of symptoms induced by PHGS may be effectively shortened by acyclovir treatment given in the first 72–96 h of onset [14, 15]. However, viral culture would usually take up to 2 weeks to confirm the diagnosis. Therefore, to clearly define the clinical features of PHGS in children would be important to make a precise diagnosis of these patients for the appropriate initial treatment.

## Methods

### Ethics statement

The study proposal was reviewed and approved by the Institutional Review Board of Chang Gung Memorial Hospital in 2017 (IRB No.201700757B0). Clinical information was collected and analyzed retrospectively and anonymously; therefore, an informed consent was waived.

### Study design and population

We conducted this retrospective study in Chang Gung Memorial Hospital (CGMH), which provides from primary to tertiary care and is the largest tertiary medical center in Taiwan. In CGMH, clinical pediatricians would usually perform viral culture by obtaining a throat or nasopharynx swab for most inpatients with clinical suspicion of viral infection such as pharyngitis, upper and lower respiratory tract infection. During January 2012 and December 2016, inpatients aged less than 19 years with HSV identified in virus culture (from the virologic laboratory logbook) were enrolled. After excluding those who had > two kinds of viruses identified or had positive bacterial culture, a total of 282 pediatric inpatients were included. Two medical students and one pediatrician reviewed their medical records retrospectively. Demographic data, clinical symptoms, laboratory results and clinical outcomes were collected and analyzed. Only those with oral manifestations such as pharyngitis, stomatitis (ulcers, vesicles, erosions) or gingivitis were considered as probable primary herpetic gingivostomatitis (PHGS) and included for final analysis.

### Viral isolation and identification

The clinical specimens of throat swab, nasopharyngeal swab, sputum, bronchoalveolar lavage (BAL) or pleural effusion were inoculated into human embryonic lung cells (HEL), rhabdomyosarcoma cells (RD) and Madin-Darby canine kidney cells (MDCK) while those of blood, cerebrospinal fluid (CSF), urine, stools or rectal swabs were inoculated into adenocarcinoma human alveolar basal epithelial cells (A549), monkey kidney cells (MK-2), RD cells and HEL cells. Cultures were maintained in minimal essential media containing antibiotics, incubated at 33 C, and rotated at 12 revolutions per hour. The inoculated cell cultures were maintained and observed for the presence of cytopathic effects (CPE) for at least 4 weeks. Respiratory viruses, including human adenovirus (HAdV), human metapneumovirus, human parainfluenza virus types 1, 2, and 3, influenza A and B viruses, Cytomegalovirus, HSV-1, HSV-2 and respiratory syncytial virus in CPE-positive cases were further identified by indirect immunofluorescence assay (IFA) with D3 Ultra DFA respiratory virus screening and identification kit (Diagnostic Hybrids, Inc., Athens, OH, USA). Anti-HSV 1 monoclonal antibody (ARGENE) react with cells infected with herpes simplex type 1 and display clear staining with Fluorescein (FITC)-conjugated AffiniPure Goat Anti-Mouse IgG + IgM (H + L) (Jackson).

### Definitions

We classified the cases of probable PHGS into three groups, namely early diagnosis, late diagnosis and other diagnosis groups. Patients diagnosed with PHGS at the time of admission were classified as the Early Diagnosis group. Those diagnosed as other diseases at admission but eventually diagnosed with PHGS before discharge from the hospital were classified as the Late Diagnosis group. Those not diagnosed with PHGS throughout hospitalization were classified as the Other Diagnosis (than PHGS) groups.

Clinical laboratory data on admission and the peak or nadir values during hospitalization were collected for analysis. We defined white blood cell count (WBC) above 15,000/μL as leukocytosis while those below 5000/μL were defined as leukopenia. A platelet count above 450,000/μL was defined as thrombocytosis while that below 150,000/μL were defined as thrombocytopenia. A serum creatinine (Cr) level above 1.0 mg/dL was considered to be renal function impairment while a 3-fold elevation of the normal values of aspartate aminotransferase (AST) and alanine aminotransferase (ALT) were considered to be elevated liver enzyme levels.

### Statistical analysis

We used the Chi-square test or Fisher’s exact test to compare categorical variables. Non-categorical variables were compared by one-way independent analysis of variance with post-hoc analysis. Data analyses were performed using SPSS software version 20.0 (SPSS Inc., Chicago, IL, USA). A *p*-value < 0.05 was considered statistically significant.

## Results

Of the 282 herpes simplex virus isolates, all were identified as herpes simplex virus type 1 (HSV-1). After excluding 97 cases without oral manifestations, a total of 185 probable cases of PHGS were included for final analysis. The specimens positive for HSV were obtained by throat swab (182/185, 98.4%), nasopharynx (1/185, 0.5%), and oral ulcer wound (2/185, 1%). Sixty-five patients (35%) were diagnosed with PHGS at the time of admission (Early Diagnosis group), 74 patients were diagnosed during hospitalization (Late Diagnosis group) and 46 (25%) patients were not diagnosed with PHGS throughout the hospitalization (Other Diagnosis group). Among the patients in Late and Other Diagnosis groups, most of the patients (60/120, 50%) were clinically diagnosed with herpangina or hand, foot, and mouth disease (HFMD) (due to enterovirus infection), followed by acute tonsillitis (35.8%) at the time of admission. The detailed clinical diagnoses are listed in Table 1. In the Late Diagnosis group, the mean interval from admission to the diagnosis of PHGS was 2.4 ± 1.42 days, with the longest of 6 days.

**Table 1:** Clinical features of 185 pediatric inpatients with culture-confirmed herpetic gingivostomatitis

Characteristics	Total No. (%)	Late Diagnosis No. (%)	Early Diagnosis No. (%)	Other Diagnosis No. (%)	*P*-value
Case Number	185	74	65	46	
Gender					0.053
Male	92 (49.7)	33 (44.6)	29 (44.6)	30 (65.2)	
Female	93 (51.3)	41 (55.4)	36 (55.4)	16 (34.8)	
Age, mean + SD (years)^b^	3.85 ± 3.58	3.23 ± 3.01	3.52 ± 3.14	5.33 ± 4.57	0.005
< 3 years	112 (60.5)	50 (67.6)	42 (64.6)	20 (43.5)	0.023
> 5 years	46 (24.9)	14 (18.9)	17 (26.2)	15 (32.6)	0.231
Diagnosis at admission					
Herpangina/HFMD	60 (32.4)	41 (55.4)	NA	19 (41.3)	0.241
Acute tonsillitis/pharyngitis	43 (23.3)	21 (28.4)	NA	22 (47.8)	0.03
LRTI^a^	7 (3.8)	4 (5.4)	NA	3 (6.5)	> 0.9
FWLS	3 (1.6)	3 (4.1)	NA	0	0.284
Others	7 (3.8)	5 (6.7)	NA	2 (4.4)	0.706
Diagnosis at discharge					
Herpangina/HFMD	20 (10.8)	NA	NA	20 (43.5)	NA
Acute tonsillitis/pharyngitis	22 (11.9)	NA	NA	22 (47.8)	NA
LRTI	4 (2.2)	NA	NA	4 (8.7)	NA
Others	0	NA	NA	0	NA
General symptoms					
Fever	184 (99.5)	74 (100)	64 (98.5)	46 (100)	0.395
Fever> 39°c	133 (77.3)	53 (79.1)	44 (74.6)	36 (78.3)	0.819
Before admission (days)^b^	3.72 ± 2.03	3.45 ± 2.01	4.28 ± 2.25	3.34 ± 1.55	0.034
Duration, mean + SD (days) [Range]^b^	5.11 ± 2.24 [1–17]	5.26 ± 2.11 [2–14]	5.43 ± 2.61 [0–17]	4.41 ± 1.72 [1–8]	0.047
> 7 days	39 (21.1)	16 (21.6)	16 (24.6)	7 (15.2)	0.484
Decreased appetite	176 (95.1)	72 (97.3)	63 (96.9)	41 (89.1)	0.092
Decreased activity	131 (70.8)	55 (74.3)	42 (64.6)	34 (73.9)	0.394
Dehydration	155 (84.7)	59 (80.8)	57 (89.1)	39 (84.8)	0.409
Mild	142 (91.6)	56 (94.9)	54 (94.7)	32 (82.1)	0.045
Moderate	13 (8.4)	3 (5.1)	3 (5.3)	7 (17.9)	0.045
Respiratory symptoms					
Cough	71 (38.4)	28 (37.8)	20 (30.8)	23 (50)	0.121
Rhinorrhea	65 (35.1)	30 (40.5)	20 (30.8)	15 (32.6)	0.445
Nasal congestion	16 (8.6)	7 (9.5)	4 (6.2)	5 (10.9)	0.650
Conjunctivitis	4 (2.2)	2 (2.7)	1 (1.5)	1 (2.2)	0.892
Extra-pulmonary symptoms					
Vomiting	36 (19.5)	12 (16.2)	10 (15.4)	14 (30.4)	0.094
Diarrhea	22 (11.9)	11 (14.9)	8 (12.3)	3 (6.5)	0.387
Abdominal pain	14 (7.6)	10 (13.5)	1 (1.6)	3 (6.5)	0.027
Skin rash	16 (8.6)	11 (14.9)	2 (3.1)	3 (6.5)	0.036
Trunk	10 (62.5)	5 (45.5)	2 (100)	3 (100)	0.049
Extremities	10 (62.5)	7 (63.6)	1 (50)	3 (100)	0.262
Face	7 (43.8)	5 (45.5)	1 (50)	1 (33.3)	0.914
Whitlow	1 (6.3)	1 (9.1)	0	0	0.677
Complications	4 (2.2)	2 (1.4)	2 (4.7)	0	0.159
CNS	4	2	2	0	0.329
PICU admission	4	2	2	0	0.159
Mortality	0	0	0	0	NA
Antibiotics treatment (> 3 days)	41 (22.2)	14 (18.9)	7 (10.7)	20 (43.4)	< 0.001
Acyclovir treatment	93 (50.3)	50 (67.6)	42 (64.6)	1 (2.2)	< 0.001
LOS (days)^b^	4.54 ± 1.92	4.97 ± 1.81	4.26 ± 2.15	4.22 ± 1.62	0.039

Abbreviations: *HFMD* Hand-foot-mouth disease, *FWLS* Fever without localizing sign, *CNS* Central nervous system, *PICU* Pediatric intensive care unit, *LOS* Length of hospital stay

^a^*LRTI* Lower respiratory tract infection, including pneumonia, bronchopneumonia and acute bronchiolitis

^b^the number presented in these items did not represent No. (%)

### Demographic and clinical characteristics

Median age of the patients was 2 years, ranging from 7 months to 17 years. The mean age of children in the Other Diagnosis group was significantly older than those in the other two groups (*p*-value = 0.005). Detailed clinical characteristics of the patients are shown in Table 1. All but one patients had fever and 77.3% had a fever higher than 39 °C. The mean duration of fever was 5.11 days and 39 patients (21%) had a fever > 7 days. Children in the Other Diagnosis group had a significantly shorter duration of fever than those in the other two groups (*p*-value = 0.047).

Oral ulcer (84.3%) was the most common oral manifestation, which was equally resided in the anterior and posterior oral cavity, followed by gum swelling or erythema and sore throat. The detailed oral manifestations stratified by the different time points of hospitalization are shown in Table 2. At the time of admission, children in the Early Diagnosis group were significantly more likely to have inflammation change over gingiva (76.2%) and ulcers over the anterior part of oral cavity (76.9%) than those in the other two groups (both *p*-value < 0.001). During the course of hospitalization, oral manifestations increased significantly in children in the Late Diagnosis group and the differences of oral manifestations between the Early and Late Diagnosis groups became statistically insignificant. In contrast, compared with those in the other two groups, children in the Other Diagnosis group were significantly more likely to have exudate coated on the tonsils and significantly less likely to have gum swelling/bleeding and oral ulcers (all *p*-value < 0.01). Meanwhile, oral ulcers in the Other Diagnosis group tended to reside in the posterior oral cavity.

**Table 2:** Detailed oral manifestations at different timelines of hospitalization

Characteristics	Total No. (%)	Late Diagnosis No. (%)	Early Diagnosis No. (%)	Other Diagnosis No. (%)	*P*-value
Case Number	185	74	65	46	
Symptoms					
Sore throat	91 (49.2)	40 (54.1)	23 (35.4)	28 (60.9)	0.017
Drooling	23 (12.4)	9 (12.2)	12 (18.5)	2 (4.3)	0.085
Signs					
Ulcers	156 (84.3)	69 (93.2)	61 (93.8)	26 (56.5)	< 0.001
Anterior oral cavity	121 (65.4)	55 (74.3)	56 (86.2)	10 (21.7)	< 0.001
Posterior oral cavity	117 (63.2)	56 (75.7)	36 (55.4)	25 (54.3)	0.016
Gum swelling/bleeding	125 (67.6)	59 (79.7)	60 (92.3)	6 (13.0)	< 0.001
Exudate coated of tonsils	31 (16.8)	12 (16.2)	4 (6.2)	15 (32.6)	0.001
On admission					
Ulcers					
Anterior oral cavity	82 (44.3)	22 (29.7)^a^	50 (76.9)	10 (21.7)	< 0.001
Posterior oral cavity	102 (55.1)	45 (60.8)	33 (50.8)	24 (52.2)	0.443
Gum swelling/bleeding	57 (31.1)	7 (9.5)^b^	48 (76.2)	2 (4.3)	< 0.001
Exudate coating	30 (16.2)	12 (16.2)	4 (6.2)	14 (30.4)	0.003
During hospitalization					
Ulcers					
Anterior oral cavity	121 (65.4)	55 (74.3)^a^	56 (86.2)^c^	10 (21.7)	< 0.001
Posterior oral cavity	117 (63.2)	56 (75.7)	36 (55.4)	25 (54.3)	0.016
Gum swelling/bleeding	125 (67.6)	59 (79.7)^b^	60 (92.3)^d^	6 (13.0)	< 0.001
Exudate coating	31 (16.8)	12 (16.2)	4 (6.2)	15 (32.6)	0.001

^a^Within Late Diagnosis group, the percentage of ulcers over anterior oral cavity increased significantly during course of hospitalization (*p*-value< 0.001)

^b^Within Late Diagnosis group, the percentage of gum swelling or bleeding increased significantly during course of hospitalization (*p*-value< 0.001)

^c^The difference of percentage of ulcers over the anterior oral cavity between the Early and Late Diagnosis group became insignificant during the course of hospitalization (*p*-value = 0.09)

^d^The difference of percentage of gum swelling/bleeding between the Early and Late Diagnosis group became insignificant during the course of hospitalization (*p*-value = 0.051)

### Laboratory parameters

Detailed laboratory data of the patients are shown in Table 3. Leukocytosis was noted in 52 (28.1%) children and a serum CRP level of > 40 mg/L (normal, < 5 mg/L) was found in 55 (29%) children. Seventeen patients (9.3%) had a peak serum C-reactive protein (CRP) level > 100 mg/L (mimicking bacterial infection), with the highest being 193.09 mg/L. None experienced renal function impairment and only three cases had an elevated aspartate aminotransferase level. There were no statistically significant differences among the three groups in terms of laboratory data. Among the children in the Other Diagnosis group, only 2 (4.3%) patients had a final report of virus culture results before discharge from the hospital.

**Table 3:** Laboratory findings of 185 inpatients with culture confirmed herpetic gingivostomatitis

Laboratory data	Total	Late Diagnosis	Early Diagnosis	Other Diagnosis	*P*-value
Case Number	185	74	65	46	
Hemogram					
WBC count (1000/μL)^a^	12.44 ± 4.95	13.05 ± 5.35	12.43 ± 4.77	11.46 ± 4.44	0.229
WBC count-peak^b^	12.47 ± 4.89	13.12 ± 5.31	12.41 ± 4.65	11.49 ± 4.4	0.209
WBC count-nadir	10.64 ± 4.01	10.96 ± 3.70	10.98 ± 4.05	9.62 ± 4.34	0.147
< 5000/μL (No. (%))	6 (3.2)	1 (1.4)	2 (3.1)	3 (6.5)	0.315
> 15,000/μL (No. (%))	52 (28.1)	23 (31.1)	19 (29.2)	10 (21.7)	0.525
Platelet (1000/μL)	251.72 ± 73.0	260.57 ± 66.42	253.25 ± 64.26	235.37 ± 91.25	0.181
< 150,000/μL (No. (%))	10 (5.4)	3 (4.1)	2 (3.1)	5 (10.9)	0.167
> 450,000/μL (No. (%))	4 (2.2)	1 (1.4)	0	3 (6.5)	0.057
Biochemistry					
CRP (mg/L)^a^	40.31 ± 37.47	40.00 ± 40.24	35.52 ± 34.68	47.48 ± 36.31	0.256
CRP-peak (mg/L)^b^ [Range]	41.86 ± 38.15 [1.3–193.09]	40.51 ± 40.01 [2.1–193.09]	37.78 ± 36.73 [1.3–165.97]	49.7 ± 36.7 [1.4–132.7]	0.252
< 40 mg/L (No. (%))	130 (71.0)	51 (69.9)	48 (75)	31 (67.4)	0.659
> 100 mg/L (No. (%))	17 (9.3)	8 (11.0)	4 (6.2)	5 (10.9)	0.564
BUN (mg/dL)	8.8 ± 2.83	9.05 ± 3.09	8.67 ± 2.26	8.61 ± 3.07	0.876
Cr (mg/dL)	0.14 ± 0.35	0.07 ± 0.25	0.08 ± 0.28	0.31 ± 0.47	0.006
> 1.0 mg/dL (No. (%))	0	0	0	0	NA
AST (U/L) [Range]	38.39 ± 42.1 [15–434]	44.18 ± 56.49 [16–434]	32.4 ± 8.72 [17–58]	41.00 ± 40.62 [15–246]	0.369
> 3-folds normal value (No. (%))	3 (2.6)	2 (3.6)	0	1 (6.2)	0.32
Sugar (mg/dL)	94.61 ± 33.46	90.07 ± 15.31	89.36 ± 16.84	108.5 ± 58.88	0.06
Hypoglycemia (< 60) (No. (%))	1 (1.2)	1 (2.5)	0	0	0.603

^a^First laboratory exam on admission

^b^The highest or lowest laboratory value during hospitalization

Abbreviations: *WBC* White blood cell, *Hb* Hemoglobin, *CRP* C-reactive protein, *BUN* Blood urea nitrogen, *Cr* Creatinine, *AST* Aspartate aminotransferase

### Clinical outcomes and complications

Forty-one (22.2%) cases received antibiotics treatment for more than 3 days during the hospitalization. Compared with those in the other two groups, children in the Early Diagnosis group were significantly less likely to receive antibiotic treatment (16.9 vs. 36.7%, *p*-value = 0.009). Half of the 185 cases received acyclovir treatment during hospitalization. The rate of acyclovir usage was significantly lower for patients in the Other Diagnosis group (2.2%) than in the Late Diagnosis group (67.6%), and in the Early Diagnosis group (64.6%) (Table 1). No statistically significant difference was found between the children receiving and not receiving acyclovir treatment in terms of duration of fever (5.20 ± 2.38 vs. 5.01 ± 2.10, *p*-value = 0.295) and length of hospital stay (4.76 ± 2.06 vs. 4.30 ± 1.74, *p*-value = 0.17). Early usage of acyclovir within 72 h of disease onset did not have benefit in reducing the duration of fever or length of hospital stay, either (Table 4).

**Table 4:** Effectiveness of acyclovir treatment between different time points

	< 72 h	> 72 h	Not used	*P*-value
Case number (%)	43 (23.2)	51 (27.6)	91 (49.2)	
Fever duration (days)	5.21 ± 2.82	5.24 ± 1.96	4.99 ± 2.11	0.778
LOS (days)*	4.58 ± 2.32	4.96 ± 1.82	4.27 ± 1.73	0.121

^*^Length of hospital stay

Four patients in this series had central nervous system (CNS) dysfunction (Table 1). An 18-month-old girl presented with status epilepticus for more than 30 min on day 2 of fever onset. One 7-year-7-month-old boy presented visual hallucinations on day 3 of fever onset. Both of them were considered as encephalitis/encephalopathy due to enterovirus infection initially. Magnetic Resonance Imaging (MRI) was performed on them and there were no documented abnormal findings. The other 2 cases were diagnosed with PHGS at the time of admission but presented symptoms of CNS dysfunction during hospitalization. The first one was an 11-year-old boy who developed conscious disturbance later and the second one was a 2-year-old girl who had frequent seizure attacks on day 5 of disease onset. Electroencephalogram (EEG) examination was performed in all of them, but none had abnormal focal epileptic form discharge. Only one case received spinal tapping, and cerebrospinal fluid studies showed no pleocytosis and negative for HSV by polymerase chain reaction and viral culture. None of them died, had neurologic sequelae and required long term usage of anticonvulsants.

## Discussion

Results from this study showed that fever remained the most common clinical symptom in children with PHGS. The duration of fever in children with other virus-induced diseases usually ranged from two to 4 days, while children with PHGS would suffer for a longer fever duration [16, 17]. In this study, the average fever duration was 5 days and more than 20% of the patients even had a fever duration longer than 7 days.

In this series, only 35% of the cases were diagnosed with PHGS at the time of admission. Most of the cases not diagnosed early were considered to be herpangina/HFMD (due to enteroviral infection) initially. In general pediatric practice, it is believed that HSV-related infection would cause vesicles and/or ulcers in the perioral region and anterior oral cavity [18–20]. On the contrary, the oral lesions of herpangina and HFMD typically located at the posterior oral cavity region, such as the anterior pillars of the fauces, soft palate, tonsils, and uvula, presented as hyperemic vesicles and/or ulcers. However, coxsackievirus A6, a specific serotype of enterovirus, could result in so-called atypical HFMD with clinical manifestations of perioral vesicles and more devastating skin lesions such as onychomadesis and even Stevens-Johnson syndrome since 2008 [21, 22]. In this series, we found the oral ulcers in PHGS could be equally resided in both the anterior and posterior parts of oral cavity. Compared with the patients in the Early Diagnosis group, the patients in both the Late and Other Diagnosis groups had a significantly lower rate of the presentation of both stomatitis lesions over the anterior oral cavity and gingivitis at the time of admission. The presentation of both lesions reached a comparable rate during hospitalization in the patients in the Late Diagnosis group while still significantly lower in the patients in Other Diagnosis group. These findings suggest that both the stomatitis lesions over the anterior part of the oral cavity and gingivitis are key features for a clinical diagnosis of PHGS. A case of PHGS may manifest stomatitis lesions over the posterior part of the oral cavity first, which may be misdiagnosed as herpangina caused by enterovirus, and then the stomatitis lesions may extend to the anterior part of oral cavity and the presentation of gingivitis may occur, which leads to the diagnosis of PHGS. For those without the presentation of both lesions, a clinical diagnosis of PHGS would be not considered and not made on most occasions. It is not impossible that some cases might indeed present both lesions but were missed by the clinicians and subsequently undiagnosed.

Only a few cases of PHGS in this series had complications such as CNS dysfunction, compared to certain serotype of enterovirus, such as enterovirus D68 and enterovirus A71, which may lead to fatal viral meningitis or encephalitis and result in limb paralysis or mortality [23, 24].

C-reactive protein (CRP), a laboratory test, is often used as a reference biomarker to distinguish bacterial (higher level) from viral (lower level) infections, though inconclusively. Nevet et al [25] reported that a high value of CRP was commonly seen in pediatric patients with PHGS and the average value of 66 patients in their study was up to 74 mg/L, with more than one-third of the patients having a value > 70 mg/L. In the current study, along with leukocytosis, a higher serum CRP level > 40 mg/L (normal, < 5 mg/L) was found in around 30% of the cases. High values of CRP are prevalent in patients with primary herpetic gingivostomatitis, similar to adenoviral infections and some bacterial infections. In contrast, for enteroviral infection, a retrospective study including 3566 children with a diagnosis of either herpangina or HFMD in Taiwan revealed that three quarters of the cases had a CRP level < 40 mg/L while only 6% cases had a CRP level > 80 mg/L [26]. CRP levels in patients infected with different serotypes of enteroviruses may vary from a normal level to a higher level. For example, an average CRP level was 9 mg/L for children with enterovirus-A71 infection while the average level was up to 77 mg/L for children infected with coxsackievirus A2 [27]. Other studies also showed the different CRP levels in children infected with different enterovirus serotypes [21, 28–30]. These findings suggest that a CRP level is not reliable to distinguish PHGS from both bacterial and enteroviral pharyngitis.

Some studies indicated that administration of oral acyclovir within 72 to 96 h after disease onset can effectively reduce the duration of fever, oral ulcers, and food intake difficulty in children with PHGS [31–33]. Half of the cases in this study received acyclovir treatment. However, the administration of acyclovir treatment, whether within 72 h of fever onset or not, had no significant beneficial influence on either the duration of fever or the length of hospital stay. Since this was a retrospective study, the exact timing and dosing of acyclovir therapy as well as the healing of oral ulcers and the amount of food intake might not be meticulously evaluated and recorded and subsequently led to a negative result of the effect of acyclovir therapy.

In this series, 41 (22.2%) patients, especially those in the Other Diagnosis group, received antibiotic treatment for more than 3 days during hospitalization, though there was no evidence of bacterial infection. Again, judicious usage of antibiotics cannot be overemphasized. Recognizing that children with PHGS may manifest prolonged high fever, leukocytosis, and a high serum CRP level that mimics bacterial infections may help clinicians to avoid the unnecessary prescription of antibiotics.

There are several limitations in this study. First, as a retrospective study, there were “inherent” drawbacks. Not all clinical information and laboratory data could be collected by chart review and some confounding bias could not be avoided due to different judgments of clinical physicians. Second, we did not check serum IgG and IgM against HSV, which can distinguish primary infection from reactivation. However, in this study, we tried to identify and include PHGS cases for final analysis as possibly as we can. Because most primary HSV-1 infection presents with oral manifestations [34, 35], we excluded 97 cases without oral manifestations, who accounted for more than one-third of the initial HSV-positive patients and might be probable HSV-reactivated cases, for final analysis. Third, we already excluded those co-infected with other bacteria and viruses; however, throat swab for group A streptococcus was not performed for each case to exclude the possibility of such infection and the possibility of co-infection with other viruses could not be totally excluded. Since only virus isolation using tissue culture was applied in this study, the viruses which cannot present CPE in the selected cell lines, such as coronavirus, human metapneumovirus, human bocavirus etc., would be missed. This could explain that there were still some cases present with symptoms of upper respiratory tract infection in this study.

## Conclusions

Clinically, a case of primary herpetic gingivostomatitis may manifest stomatitis lesions over the posterior part of the oral cavity first, and then the lesions may extend to the anterior part of the oral cavity with the presentation of gingivitis (as gum swelling/bleeding), which are key features for a clinical diagnosis of PHGS. Meticulously identifying these specific oral manifestations can help make the diagnosis of PHGS earlier and subsequently reduce unnecessary prescription of antibiotics.

## Data Availability

The datasets used and/or analysed during the current study are available from the corresponding author on reasonable request.

## Abbreviations

HSV: Herpes simplex virus
PHGS: Primary herpetic gingivostomatitis
CRP: C-reactive protein
HFMD: Hand, foot and mouth disease
CNS: Central nervous system
